# Antagonistic effects of HTLV-1 Tax oncoprotein on BRCA1 expression and function

**DOI:** 10.1186/1742-4690-11-S1-P88

**Published:** 2014-01-07

**Authors:** Mahmoud Huleihel, Mital Shukron, Azhar Gabarin, Ammar Abukandil, Mordechai Aboud

**Affiliations:** 1 Department of Microbiology, Immunology and Genetics, Faculty of Health Sciences, Ben-Gurion University of the Negev, Beer-Sheva, Israel

## 

HTLV-1 Tax oncoprotein is considered a key factor in HTLV-1 pathogenicity. BRCA1 gene dysfunction can lead to breast cancer development. In contrast to the tumor suppressor nature of BRCA1, Tax is a potent oncoprotein, most of its activities are strictly opposing those of BRCA1. Therefore, we hypothesize that HTLV-1 Tax expression in breast epithelial cells can antagonize BRCA1 expression and functionality, thereby sensitizing these cells to malignant transformation by environmental carcinogens. Our objective was to provide molecular and cellular indications to validate this hypothesis. Based on earlier findings that the milk of HTLV-1 infected women is rich in HTLV-1 infected lymphocytes that can transfer the virus into breast epithelial cells, the outcomes of this project may point that HTLV-1 can be a risk factor for the development of breast cancer, with a substantially higher risk to women who practice long-term breastfeeding. Our results showed that Tax strongly inhibited estrogen induced activation of BRCA-1 expression in breast cells by sequestering CBP/p300 co-activators. Trying to explore the CBP/p300 associated mechanism of Tax effect on BRCA1 expression, our results suggest that Tax does not prevent the binding of CBP/p300 to ERα but rather physically associates with the ERα-CBP/p300 to form a tertiary reporter. Since CBP/p300 complex has several binding domains, we believe that Tax associates with ERα-CBP/p300 complex through binding to CBP/p300 rather than to the ERα protein. We have also found that Tax inhibits BRCA1-mediated activation of p53-target promoters. These results support the possibility of HTLV-1 involvement in breast cancer development.

